# Difference in the Surgical Outcome of Unilateral Cleft Lip and Palate Patients with and without Pre-Alveolar Bone Graft Orthodontic Treatment

**DOI:** 10.1038/srep23597

**Published:** 2016-04-04

**Authors:** Chun-Shin Chang, Christopher Glenn Wallace, Yen-Chang Hsiao, Yu-Ting Chiu, Betty Chien-Jung Pai, I-Ju Chen, Yu-Fang Liao, Eric Jen-Wein Liou, Philip Kuo-Ting Chen, Jyh-Ping Chen, M. Samuel Noordhoff

**Affiliations:** 1 Department of Chemical and Materials Engineering, College of Engineering, Chang Gung University, Taoyuan, Taiwan; 2 Craniofacial Research Center, Department of Medical Research, Department of Plastic & Reconstructive Surgery and Department of Craniofacial Orthodontics, Chang Gung Memorial Hospital, Taoyuan, Taiwan

## Abstract

Presurgical orthodontic treatment before secondary alveolar bone grafting (SABG) is widely performed for cleft lip/palate patients. However, no randomized controlled trial has been published comparing SABG outcomes in patients with, and without, presurgical orthodontic treatment. This randomized, prospective, single-blinded trial was conducted between January 2012 and April 2015 to compare ABG volumes 6 months postoperatively between patients with and without presurgical orthodontic treatment. Twenty-four patients were enrolled and randomized and 22 patients completed follow-up. Patients who had presurgical orthodontics before SABG had significantly improved inclination (p < 0.001) and rotation (p < 0.001) of the central incisor adjacent to the defect, significantly improved ABG fill volume (0.81 ± 0.26 cm^3^ at 6 months compared to 0.59 ± 0.22 cm^3^; p < 0.05) and less residual alveolar bone defect (0.31 ± 0.08 cm^3^ at 6 months compared to s 0.55 ± 0.14 cm^3^; p < 0.001) compared to patients who did not have presurgical orthodontic treatment. In conclusion, orthodontic treatment combined with SABG results in superior bone volume when compared with conventional SABG alone.

Alveolar bone grafting (ABG) is integral to the surgical management of cleft lip/palate patients. ABG can obliterate an oronasal fistula, stabilize the dental arch and offer bone matrix for adjacent dental eruption. Conversely, an unrepaired alveolar bone defect can lead to instability and increasing medial collapse of the dental arch, persistent fistula, and abnormal dental eruption either side of the defect.

Dental crowding, dental inclination towards the cleft, and rotation of the central incisor makes dental hygiene difficult for these patients, and predisposes them to plaque formation, dental caries and gingivitis. Aligning the teeth before surgery may allow improved dental hygiene, which may reduce infection and thus increase graft success.

Although preoperative orthodontic treatment is a common practice in many centers worldwide, a prospective, randomized controlled trial comparing the outcomes of secondary ABG (SABG) with, or without, pre-ABG orthodontic treatment has not previously been published. Accordingly, the purpose of this study was to determine whether preoperative orthodontic treatment affects SABG outcome.

## Results

Twelve patients in the Experimental group received orthodontic treatment before SABG and 12 patients in the Control group did not receive orthodontic treatment before SABG. Twenty-two patients completed six months of follow up after SABG, as two patients in the experimental group failed to return for postoperative 6 months follow up. Age, sex, and cleft side are tabulated (Table 1).

### Orthodontic treatment

Three patients in the Experimental group agreed to undergo pre-orthodontic CT scans. Alveolar bone defect volume before, and after, orthodontic treatment were 1.41 ± 0.26 cm^3^, and 1.10 ± 0.08 cm^3^, respectively. Inclination of the central incisor on the cleft side before orthodontic treatment was reduced by orthodontic treatment (30.51 ± 6.33 degrees versus 10.67 ± 0.50 degrees). Rotation of the central incisor adjacent to the cleft before orthodontic treatment was reduced by orthodontic treatment (41.43 ± 4.30 degrees versus 10.54 ± 1.98 degrees). Statistical significance is not reported in this group due to the small patient numbers.

### Measurements before SABG surgery

The alveolar bone defect volume before surgery for the Experimental group was similar to that of the control group (1.20 ± 0.35 cm^3^ versus 1.35 ± 0.40 cm^3^; p = 0.35). Inclination of the central incisor on the cleft side for the Experimental group was significantly less than for the Control group (9.59 ± 1.66 degree versus 24.47 ± 5.68 degree; p < 0.001). Rotation of the central incisor adjacent to the cleft for the Experimental group was significantly less than for the Control group (12.33 ± 2.66 degrees versus 48.42 ± 8.68 degrees; p < 0.001). Intra-observer consistency was high for alveolar bone defect (r = 0.91, p < 0.001), central incisor inclination (r = 0.98, p < 0.001) and central incisor rotation (r = 0.99, p < 0.001) measurements.

### Measurements six months after SABG surgery

ABG volume for the Experimental group was significantly higher than for the Control group (0.81 ± 0.26 cm^3^ versus 0.59 ± 0.22 cm^3^; p = 0.04). Residual alveolar bone defect for the Experimental group was significantly less than for the Control group (0.31 ± 0.08 cm^3^ versus 0.55 ± 0.14 cm^3^; p < 0.001). Inclination of the central incisor on the cleft side for the Experimental group was significantly less than for the Control group (6.84 ± 2.71 degrees versus 20.46 ± 5.91 degrees; p < 0.001). Rotation of central incisor adjacent to the cleft for the Experimental group was significantly less than for the Control group (8.15 ± 2.01 degree versus 41.35 ± 7.16 degree; p < 0.001). Intra-observer consistency was high for the ABG volume (r = 0.86, p < 0.001), residual alveolar bone defect (r = 0.89, P < 0.001), central incisor inclination (r = 0.99, p < 0.001) and central incisor rotation (r = 0.99, p < 0.001) measurements.

## Discussion

Presurgical orthodontic treatment is a common practice in many Craniofacial Centers to improve alignment of the inclinated and rotated central incisor adjacent to the alveolar bone cleft. To our knowledge this is the first clinical randomized controlled trial to assess the success of SABG, using CT scans, in patients with or without orthodontic treatment prior to SABG.

An alveolar bone cleft is present in the majority of patients with cleft lip and palate. This bony defect destabilizes the maxillary arch, predisposes it to medial collapse, and the impacted canine fails to erupt normally. Successful alveolar bone grafting completes and thereby stabilizes the maxillary arch^1^,^2^. Moreover, a successful ABG can normalize occlusion, provide a matrix for continued eruption of permanent teeth in this region, close a peri-alveolar oronasal fistula and provide periodontal health for teeth adjacent to the cleft.

Two-dimensional dental radiographs have been used to assess the success of ABGs for more than three decades^2^,^3^,^4^,^5^,^6^. Success was concluded based upon the measurement of bone bridges formed after bone grafting, but it has been shown that success rates can be overestimated using this methodology^7^. Two-dimensional radiographs were also unable to provide volumetric measurements. The present study used three dimensional CT (3DCT) to measure bone volume after SABG. CT measurements in the craniofacial region have been shown to match measurements on the actual object scanned, and 3DCT is not subject to the problems of image enlargement, image distortion, structural overlapping and positional problems of traditional two-dimensional radiographs^8^.

In our center, we advocate SABG around the stage of mixed dentition. Some centers prefer primary gingivoperiosteoplasy^9^, but our past experience suggested that such early intervention can cause later restriction in maxillary growth^10^. Maxillary growth and dental age were our main considerations in determining the timing of alveolar reconstruction. Maxillary growth is completed near the age of eight years old, whereas the maxillary canine does not erupt before the age of ten^11^. Therefore, to minimize maxillary growth disturbance, it has been advised that reconstruction be delayed until after growth is completed^12^. Timing alveolar bone grafting to around the stage of mixed dentition is widely accepted^5^,^13^,^14^,^15^. Performing ABG at approximately nine years of age allows the bulk of alveolar bone growth to complete and at this time the incisors are ready to erupt^16^,^17^,^18^.

The most commonly used ABG donor sites include the iliac crest^1^, calvarium^19^,^20^ and tibia^21^. Regardless of the donor site, cancellous bone is preferable to cortical or osteochondral grafts^22^. We prefer the iliac crest because it has sufficient cancellous bone to fill even a large alveolar bone defect^16^,^23^.

The orthodontists form an integral part of the cleft care team. Their recommendations regarding timing of treatment should be carefully considered before surgery. Dental crowding and malposition of the dentition around the cleft can interfere with oral hygiene. Rotation and inclination of the central incisor can predispose to plaque accumulation and increase the risk for decay and gingivitis, which can reduce alveolar bone height. Preoperative orthodontic treatment can optimize the position of the dentoalveolar structure, which enables patients to achieve better oral hygiene and prevent plaque formation prior to their operation. This can therefore prevent chronic, low grade inflammation activating proteases that degrade grafted bone^24^. Severe central inclination towards the cleft defect can also interfere with cleft muco-periosteal dissection. Less central incisor inclination therefore eases surgery, thereby improving dissection, placement of bone graft and wound closure.

Some centers have intuitively recommended that orthodontic treatment becomes part of their treatment protocol for their patients^25^,^26^. However, this is the first randomized controlled study to evaluate the role of preoperative orthodontic treatment on ABG success.

Central incisor inclination and rotation were measured on 3DCT scans. The head was first oriented along the infra-orbital plane in both coronal and axial views. The central incisor was defined, and its inclination was measured in relation to the vertical line on coronal view. Rotation of the central incisor was measured in relation to the horizontal line on axial view. These measurements are slightly different to those of another study^24^. Their measurements were based on two-dimensional radiographs and dental casts. Central incisor rotation is difficult to measure accurately on a dental cast, especially when teeth are missing or crowded, or when the inter-central-incisor midline is deviated. Three-dimensional CT allows easy visualization of dental relationships to the cranium, and it is simple and accurate to measure inclination with respect to the vertical line and rotation with respect to the horizontal line.

We have demonstrated that orthodontic treatment before SABG improves both inclination and rotation of the central incisor adjacent to the cleft. These improvements are maintained six months after surgery. The SABG volume was significantly improved and the residual alveolar bone defect was significantly reduced in the orthodontic treatment Experimental group in comparison to the Control group that did not receive presurgical orthodontic treatment.

### Limitations of the present study

This study does have limitations. Firstly, the pre-orthodontic CT scan was optional for our patients and only three parents/caregivers consented for their children to have 3DCT scans. However, by excluding all patients without pre-orthodontic CT scans would vastly increase the duration required to complete such a study with adequate power. Secondly, two patients did not complete their six months of follow-up. Thirdly, we used conventional facial CT scans instead of the potentially superior cone-beam CT scans to perform measurements, because conventional CT scans are routinely performed in our center to visualize the three-dimensional anatomy of the bony defect before surgery. Fourthly, it was not possible to perform a double-blinded study because the orthodontic appliances are unavoidably visible on the preoperative CT scans (note that they are removed prior to, and therefore not visible on, the six month follow up CT scans).

## Conclusion

Patients who received orthodontic treatment prior to SABG had improved central incisor position before surgery and improved bone graft volume in comparison to patients who received SABG without presurgical orthodontic treatment.

## Methods

This was a randomized, single-blinded, prospective, two-armed, parallel clinical trial designed to investigate whether presurgical orthodontic treatment affected the volume of SABG take.

### Ethics

This trial was approved by the institutional review board (IRB) of Chang Gung Memorial Hospital (IRB 99–3910A3 and 102–3500C) and the study methods were carried out in accordance with the approved guidelines of IRB. The date of ClinicalTrial.gov registration was 17^th^ February 2015 (NCT02454998). The date at which the ethics committee approved the study was 21^th^ April 2011, the date that patient recruitment started was 1^st^ January 2012 and the date that follow-up completed for the final patient was 30^th^ April 2015.

### Study sample size

The sample size was calculated based on the results of a pilot study. We analyzed ten consecutive cleft patients with CT scans. The average alveolar cleft defect volume was 1.05 ± 0.19 cm^3^. If a difference of bone resorption of 25% is considered significant, given the same standard deviation, power of 0.8 and alpha of 0.05, the total number of patients calculated for each group would be 12.

### Patients and randomization

Accordingly, 24 consecutive patients were enrolled to this study between January 2012 and April 2015 for randomization having satisfied the following criteria. Inclusion criteria were: (1) patients with unilateral complete cleft lip; (2) patients with alveolar bone cleft diagnosed by conventional radiography; (3) patients at the age of mixed dentition; (4) valid IRB-approved written informed consent provided. Exclusion criteria were: (1) presence of other craniofacial abnormalities; (2) consent withheld either to the study or to the procedures (SABG and/or orthodontic treatment).

**Figure 1:**
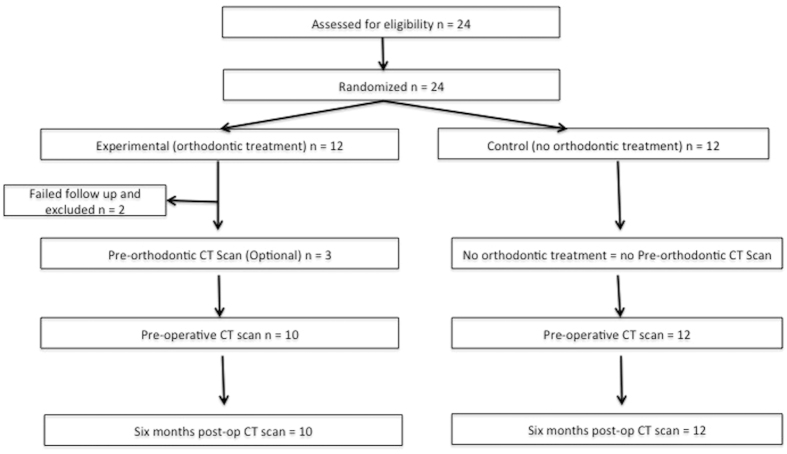
Consort statement flow chart.

These 24 patients were block randomized to a 1:1 ratio by an independent third-party specialized trials nurse into an Experimental group (received preoperative orthodontic treatment before SABG) or a Control group (received SABG alone, without preoperative orthodontic treatment) using secure randomization envelopes (Fig. 1).

### Orthodontic trneatments

Orthodontic treatments in the Experimental group were commenced 6 months before SABG by craniofacial orthodontics specialists and continued for 4 months after surgery. All such patients were treated with fixed appliances to align the rotated and inclinated central incisor adjacent to the cleft.

### Secondary alveolar bone grafting, surgical procedures

**Figure 2:**
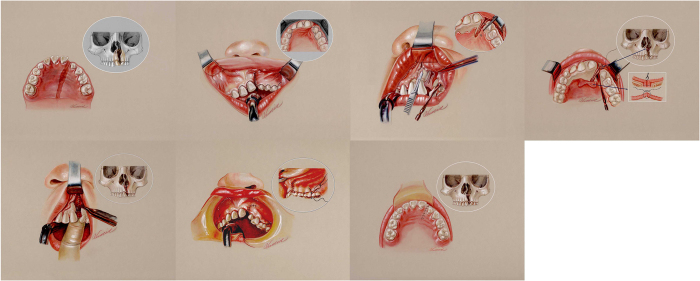
Left upper first and second: the alveolar bone defect was defined. The anatomic defect requiring alveolar bone grafting was also confirmed with three dimensional facial CT scans. Left upper third: A superiorly based gingival mucoperiosteal flap was raised to explore the alveolar bone defect. Left upper fourth: the palatal mucoperiosteal flap was raised to a level beyond the deepest margin of the alveolar fistula. Left lower first: the nasal floor tissue was completely closed. The bone graft was packed into the defect area. Left lower second and third: complete closure of gingival and palatal wounds.

Alveolar bone grafting was performed similarly to the description of Hall and Posnick (Fig. 2)^8^,^27^. Briefly, the oral cavity was cleansed with 0.1% chlorhexidine gluconate solution and the gingiva and upper buccal sulcus were infiltrated with 1% xylocaine in 1:200,000 epinephrine. Incisions were made along each side of the alveolar cleft. A superiorly based gingival mucoperiosteal flap was designed and raised sharply from the gingival margin on the lesser segment. The flap was extended posteriorly to the first molar. The incision was then curved obliquely towards the buccal sulcus. The flap on the medial segment was elevated in a similar fashion towards the midline. The palatal mucoperiosteal flaps were raised to a level beyond the deepest margin of the bucco-alveolar fistula. The fistula margins on the palatal side were freshened and sutured. Nasal floor tissue was completely separated from the palatal mucoperiosteum after raising the palatal flaps and then stripped off the bony cleft. The nasal floor tissue was dissected upwards to reach the pyriform aperture on the lateral segment and the cartilaginous septum on the medial segment. This allowed a tension-free closure of nasal floor tissue and adequate correction of the vertical discrepancy of the nostril sill. The nasal floor fistula was securely repaired with 4–0 polyglactin sutures (Vicryl, Ethicon, USA). Cancellous bone chips harvested from the right iliac crest were packed firmly into the bony defect to the level of the alveolar process and the pyriform aperture on the cleft side. The periosteum of the lateral gingival flap was scored to reduce tension especially at the lateral end of the incision. The lateral gingival flap was then advanced and sutured to the medial flap and palatal flap to provide a watertight and tension free closure (Fig. 2).

### Measurements on Three-Dimensional CT Scans and Blinding

**Figure 3:**
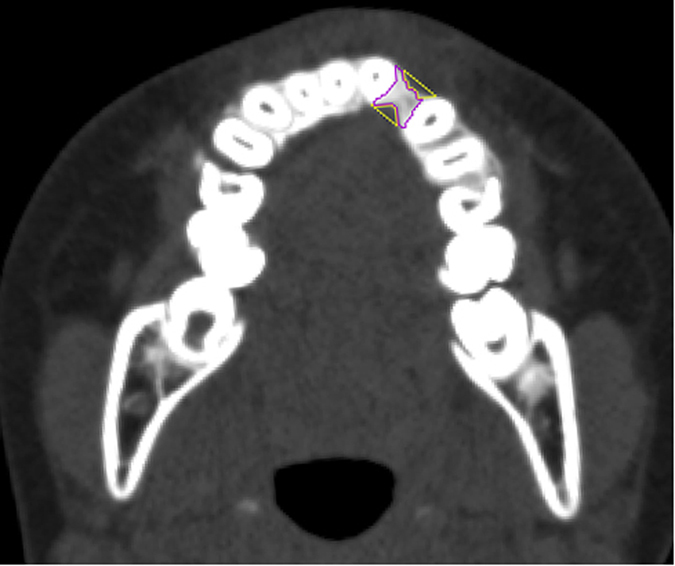
Purple: alveolar bone graft formation; Orange: Residual Alveolar Bone Defect.

**Figure 4:**
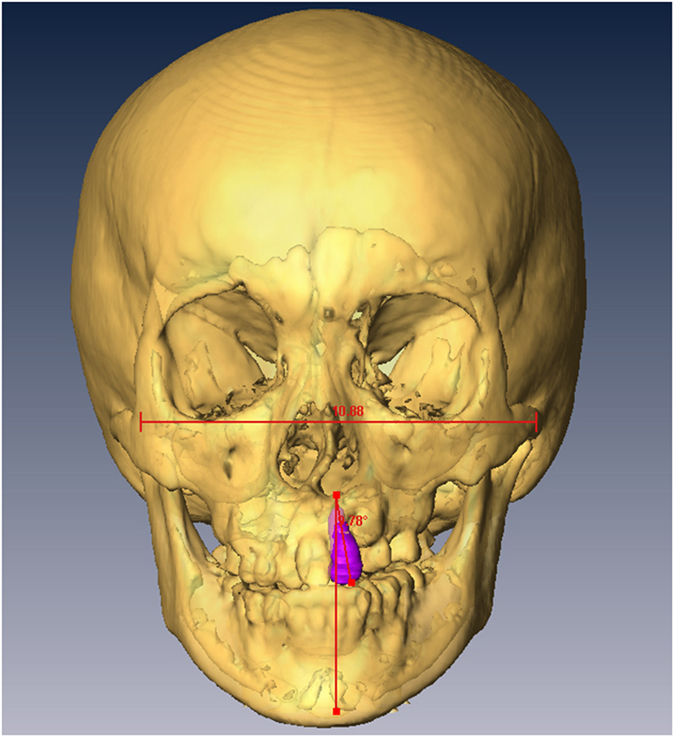
Central incisor inclination: Inclination of the central incisor was measured in degrees with respect to the vertical line.

**Figure 5:**
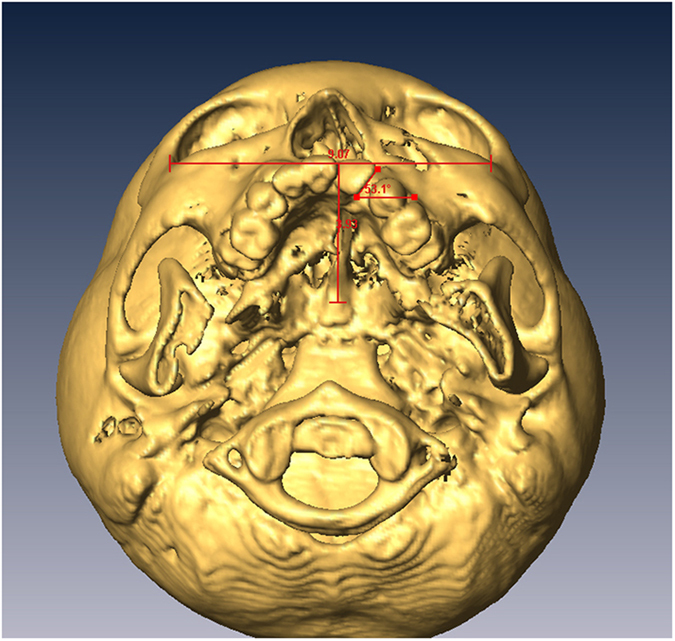
Central incisor rotation: Rotation of the central incisor was measured in degrees with respect to the horizontal line.

Computed tomography (CT) scans with 1 mm thick slices were performed before orthodontic treatments, before surgery and 6 months after surgery. CT scan data was transferred with DICOM format (Digital Imaging and Communications in Medicine) to a workstation running Avizo 7.0 (Visage Imaging, Carlsbad, CA, USA). The cranium was oriented to the Frankfort plane in the sagittal view and infraorbital line in the coronal view and axial view. The alveolar bone defect, bone graft and/or medial incisor adjacent to the cleft were defined in multiplanar imaging. Three-dimensional CT images were analyzed for: 1) alveolar bone defect; 2) alveolar bone grafting area and residual bone defect area (Fig. 3); 3) central incisor inclination (Fig. 4); and 4) central incisor rotation (Fig. 5). The assessor was blinded to the grouping of all patients, however orthodontic devices were visible on presurgical CT scans making blinding of this series of scans impossible. Blinding was achieved for the six-month follow-up scans as orthodontic appliances were not present at that time; thus, this was a single-blinded study.

### Inter- and Intra-observer consistency of assessments

Intra-observer reliability of the measurements were tested using Pearson correlation by comparing two sets of measurements performed at least one week apart by the same rater.

### Statistical analyses

All statistical analyses were conducted using SPSS software (version 17.0; IBM Corporation, NY, USA). Differences of central incisor inclination, central incisor rotation and alveolar bone defect between Experimental and Control groups were compared. Such data was measured twice, and the mean of two measurements was used for analysis using the independent t-test to compare groups. Central incisor inclination, central incisor rotation and alveolar bone defect before and after orthodontic treatment in the Experimental group were compared using the paired t-test. The Chi square test was used to compare demographic data of both groups (sex and cleft side). Statistical significance was defined if p was less than 0.05. Data are presented as mean ± standard deviation unless otherwise stated.

## Additional Information

**How to cite this article**: Chang, C.-S. *et al.* Difference in the Surgical Outcome of Unilateral Cleft Lip and Palate Patients with and without Pre-Alveolar Bone Graft Orthodontic Treatment. *Sci. Rep.* **6**, 23597; doi: 10.1038/srep23597 (2016).

## Figures and Tables

**Table 1:** Demographic data for patients.

	Experimental group	Control group	
Patient numbers	10	12	
Age (Years)	9.69 ± 0.74	9.30 ± 0.53	T test P = 0.09
Sex (Male:Female)	(3:7)	(8:4)	Chi Square P = 0.09
Cleft side (Left:Right)	(7:3)	(11:1)	Chi Square P = 0.19
Distribution of Surgeons (CSC:PKTC)	(3:7)	(5:7)	Chi Square P = 0.68
